# Baseline Gene Expression Signatures in Monocytes from Multiple Sclerosis Patients Treated with Interferon-beta

**DOI:** 10.1371/journal.pone.0060994

**Published:** 2013-04-18

**Authors:** Marta F. Bustamante, Ramil N. Nurtdinov, Jordi Río, Xavier Montalban, Manuel Comabella

**Affiliations:** Servei de Neurología/Neuroimmunología. Centre d’Esclerosi Múltiple de Catalunya, Cemcat. Hospital Universitari Vall dHebron (HUVH), Barcelona, Spain; University of Ottawa, Canada

## Abstract

**Background:**

A relatively large proportion of relapsing-remitting multiple sclerosis (RRMS) patients do not respond to interferon-beta (IFNb) treatment. In previous studies with peripheral blood mononuclear cells (PBMC), we identified a subgroup of IFNb non-responders that was characterized by a baseline over-expression of type I IFN inducible genes. Additional mechanistic experiments carried out in IFNb non-responders suggested a selective alteration of the type I IFN signaling pathway in the population of blood monocytes. Here, we aimed (i) to investigate whether the type I IFN signaling pathway is up-regulated in isolated monocytes from IFNb non-responders at baseline; and (ii) to search for additional biological pathways in this cell population that may be implicated in the response to IFNb treatment.

**Methods:**

Twenty RRMS patients classified according to their clinical response to IFNb treatment and 10 healthy controls were included in the study. Monocytes were purified from PBMC obtained before treatment by cell sorting and the gene expression profiling was determined with oligonucleotide microarrays.

**Results and discussion:**

Purified monocytes from IFNb non-responders were characterized by an over-expression of type I IFN responsive genes, which confirms the type I IFN signature in monocytes suggested from previous studies. Other relevant signaling pathways that were up-regulated in IFNb non-responders were related with the mitochondrial function and processes such as protein synthesis and antigen presentation, and together with the type I IFN signaling pathway, may also be playing roles in the response to IFNb.

## Introduction

Interferon-beta (IFNb), a first-line disease modifying therapy for relapsing-remitting multiple sclerosis (RRMS), has demonstrated beneficial effects on reducing clinical and radiological disease activity [1], [2], [3]. Nevertheless, a relatively important percentage of MS patients do not respond to IFNb [4]. Previous studies performed by our group revealed a baseline type I IFN signature in peripheral blood mononuclear cells (PBMC) from a subgroup of IFNb non-responders selected by stringent clinical response criteria after two years of treatment [5]. A type I IFN dysregulation has also been observed in hepatitis C patients who do not respond to IFN-alpha, another type I IFN [6]. Similar to the findings observed in MS non-responders to IFNb, hepatitis C non-responders present higher levels of type I IFN responsive genes such as *MX1*, *OAS1* and *STAT1* before treatment initiation [6]. Furthermore, an up-regulation of the type I IFN pathway has also been reported in other autoimmune disorders such as rheumatoid arthritis [7], systemic lupus erythematosus [8], and Sjögren’s syndrome [9].

Additional mechanistic experiments conducted in RRMS patients treated with IFNb suggested a selective alteration of the type I IFN signaling pathway in blood monocytes from IFNb non-responders based on the findings of an increased expression of the membrane IFN receptor 1 (IFNAR1) and intracellular STAT1 phosphorylation in CD14+ cells from this subgroup of patients [5]. In a later study [10], we observed that the baseline increased expression of IFNAR1 was also present in monocytes from IFNb non-responders selected according to less stringent response criteria (intermediate responders).

The biological relevance of the type I IFN pathway in monocytes and its relationship with MS pathogenesis and IFNb treatment has been underscored in recent studies [11], [12]. Conditional genetic knockout of *ifnar1* in monocytes, but not in other cell subsets, led to enhanced disease severity in the animal model of MS, experimental autoimmune encephalomyelitis (EAE) [11]. In another study, IFNb treatment in MS patients was found to induce a specific over-expression of the proapoptotic and IFN inducible gene *TRAIL* only in the monocyte and granulocyte populations among nine peripheral blood cell subsets investigated [12].

The purpose of the present study was (i) to investigate whether the type I IFN signature is observed in blood monocytes isolated from IFNb non-responders at baseline; and (ii) to identify additional biological pathways in monocytes that may be playing relevant roles in the response to IFNb treatment.

## Results

A total of 2241 genes were differentially expressed with p-values<0.05 between IFNb responders and non-responders. Of these, 1024 genes were up-regulated and 1217 down-regulated in monocytes from IFNb non-responders (see Table S1). When applying a more strict p-value threshold of 0.01, 222 genes were up-regulated and 207 down-regulated. In order to identify relevant biological pathways in monocytes from IFNb responders and non-responders, differentially expressed genes with p-values<0.01 were analyzed with the Ingenuity Pathway Analysis software.

### The Type I interferon Signaling Pathway is Over-expressed at Baseline in Monocytes from IFNb Non-responders

As shown in Table 1, 5 canonical pathways were found to be up-regulated and 1 down-regulated with p-values<0.01 in monocytes from IFNb non-responders versus responders. Less significant pathways are shown as Table S1.

**Table 1:** Pathways classified by the Ingenuity Pathways Analysis software that were differentially expressed in monocytes between non-responders and responders to IFNb treatment (p<0.01).

Top canonical pathways	P-values	Expression
Interferon signaling	0.001	Up-regulated
Mitochondrial Dysfunction	0.0012	Up-regulated
Protein Ubiquitination Pathway	0.0014	Up-regulated
Regulation of eIF4 and p70S6KSignaling	0.0022	Up-regulated
eIF2 Signaling	0.0085	Up-regulated
Molecular Mechanisms ofCancer	0.0079	Down-regulated

Up-regulated and down-regulated means that a higher percentage of genes of these pathways were up-regulated or down-regulated in non-responders versus responders.

The most differentially expressed canonical pathway corresponded to the type I interferon signaling pathway, which was up-regulated in monocytes from non-responders (p = 0.0010; Table 1). These results were further supported by the finding of STAT1, the main mediator of the signaling of IFNs, as one of the transcription factors that was over-expressed in monocytes from IFNb non-responders compared with responders (Table S1).

**Figure 1:**
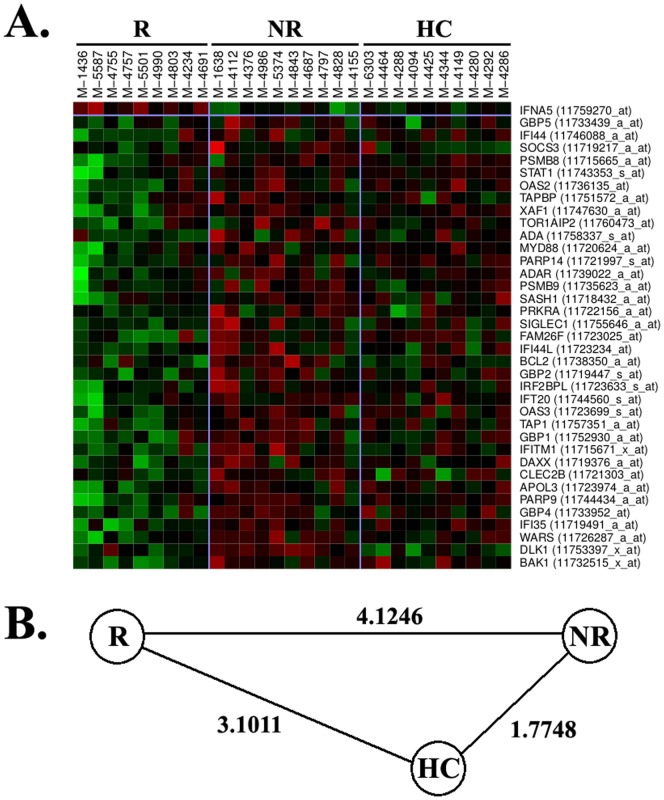
Heatmap illustration of type I IFN inducible genes. (A) The heatmap shows genes that belong to the type I IFN signaling pathway and were differentially expressed between IFNb responders and non-responders. Gene names and Affymetrix probe sets are shown on the right part of the figure. Green colour means down-regulation and red colour up-regulation. R: responders. NR: non-responders. HC: healthy controls. (B) Euclidean distance graph summarizing distances between mean expression values for all the genes displayed in the heatmap for all pairs of the study groups (R, NR, and HC).

Figure 1A shows a heatmap representation of the type I IFN responsive genes that were significantly differentially expressed in monocytes from IFNb non-responders compared with responders. The vast majority of type I IFN responsive genes were over-expressed in IFNb non-responders when compared to responders. Interestingly, the expression pattern observed for these genes in monocytes from healthy controls was more similar to IFNb non-responders than to responders (Figure 1B).

### Monocytes from IFNb Non-responders at Baseline are Characterized by an Over-expression of Mitochondria-related Genes

When exploring other biological pathways that could be playing roles in the response to IFNb, we observed that the second most differentially canonical pathway was mitochondrial dysfunction (p = 0.0012; Table 1), which was up-regulated in monocytes from IFNb non-responders.

**Figure 2:**
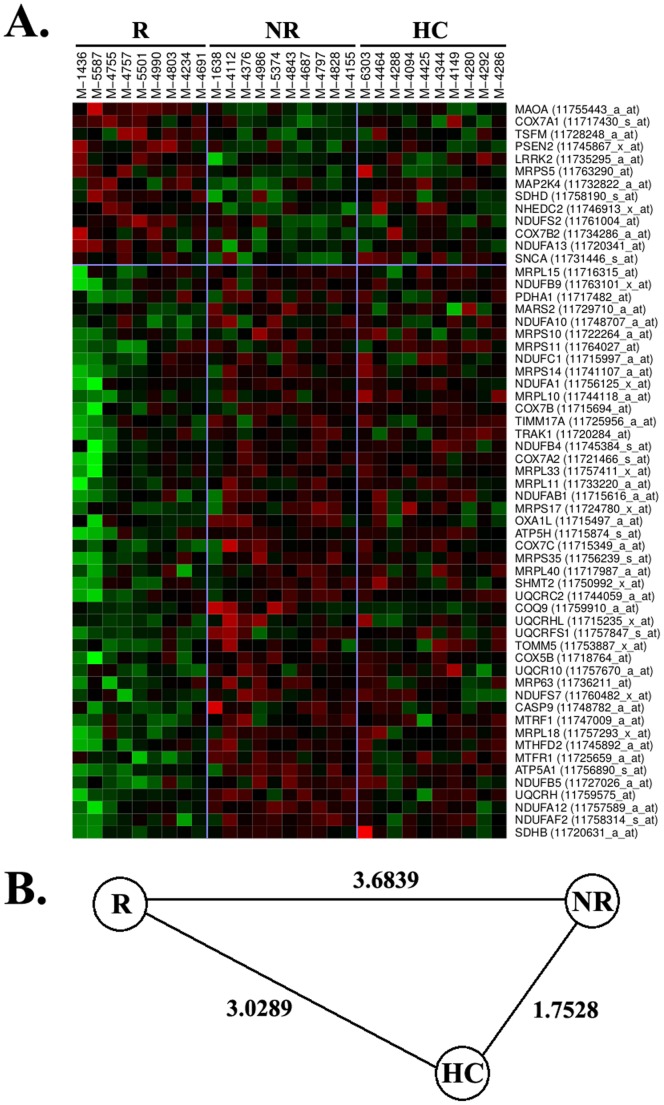
Heatmap representation of mitochondria-related genes. (A) The heatmap illustrates genes belonging to the mitochondrial dysfunction that was differentially expressed between IFNb responders and non-responders. Gene names and Affymetrix probe sets are shown on the right part of the figure. Green colour means down-regulation and red colour up-regulation. R: responders. NR: non-responders. HC: healthy controls. (B) Euclidean distance between R, NR, and HC.

As illustrated in the heatmap of Figure 2A, many of the differentially expressed genes belonged to different complexes of the mitochondrial respiratory chain and a high proportion of these genes (46 out of 59 genes) were significantly up-regulated in monocytes from non-responders. Again, the expression patterns observed for these genes in healthy controls were closer to IFNb non-responders than to responders (Figure 2B).

### Other Biological Pathways in Monocytes Potentially Involved in the Response to IFNb

As shown in Table 1, other top canonical pathways that were differentially expressed in monocytes from responders and non-responders were the EIF2 signaling (p = 0.0085), regulation of eIF4 and p70S6K signaling (p = 0.0022), and protein ubiquitination (p = 0.0014) pathways, all of which were also up-regulated in monocytes from IFNb non-responders when compared to responders.

**Figure 3:**
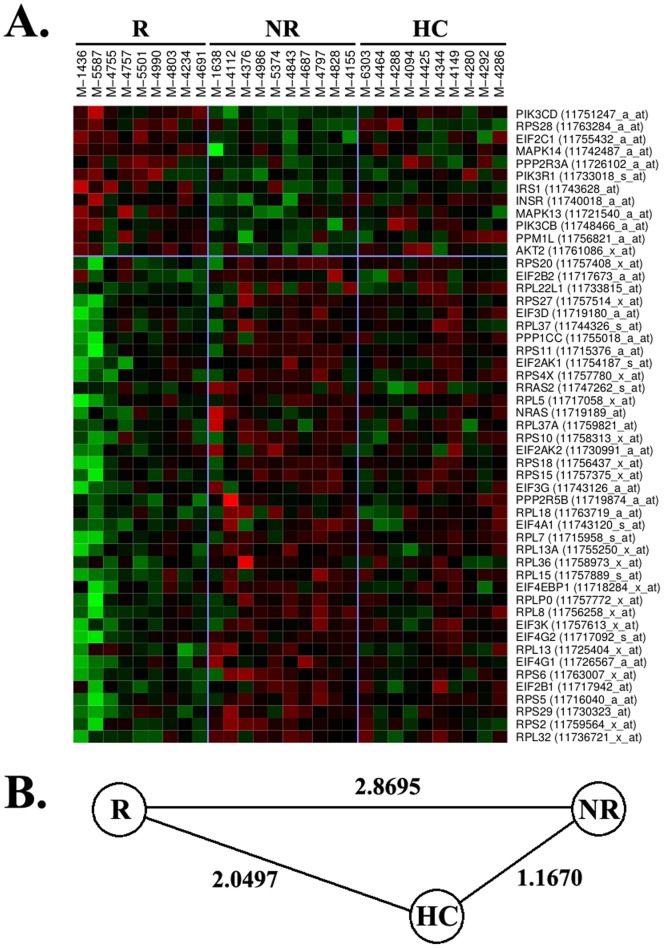
Heatmap representation of ribosomal genes. (A) The heatmap depicts ribosomal genes (eIF2 signaling and eIF4 and p70S6K signaling pathways) that were differentially expressed between IFNb responders and non-responders. Gene names and Affymetrix probe sets are shown on the right part of the figure. Green colour means down-regulation and red colour up-regulation. R: responders. NR: non-responders. HC: healthy controls. (B) Euclidean distance between R, NR, and HC.

The identification of the eIF2 signaling and eIF4 and p70S6K signaling pathways suggest an implication of ribosomes in the response to IFNb. In fact, most of the significantly differentially expressed genes belonging to these pathways were related with the protein synthesis machinery or coded for structural constituents of ribosomes (Table S2). Thirty-nine of 51 differentially expressed genes were over-expressed in monocytes from IFNb non-responders compared with responders (Figure 3A).

**Figure 4:**
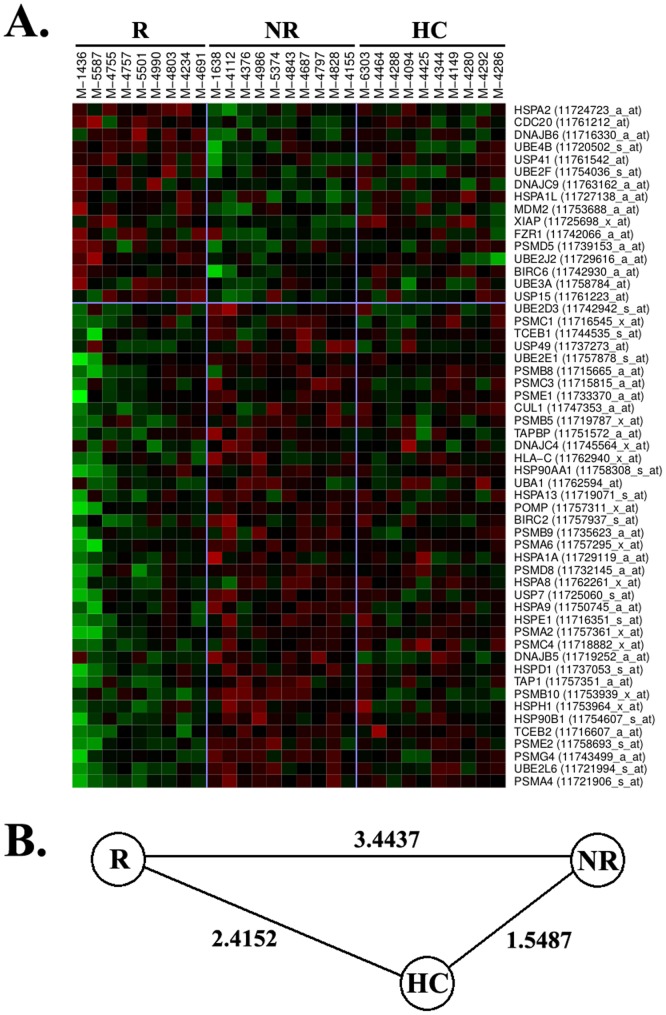
Heatmap illustration of immunoproteasome genes. (A) The heatmap shows immunoproteasome genes (protein ubiquitinitation pathway) that were differentially expressed in monocytes from IFNb responders and non-responders. Gene names and Affymetrix probe sets are shown on the right part of the figure. Green colour means down-regulation and red colour up-regulation. R: responders. NR: non-responders. HC: healthy controls. (B) Euclidean distance between R, NR, and HC.

Another pathway differentially expressed in monocytes from responders and non-responders to IFNb was protein ubiquitination. As shown in Table S2, genes included in this pathway were related to proteasome structure. Of note, these genes are components of the immunoproteasome but not of the common proteasome structure, and 39 out of 55 differentially expressed genes were found to be up-regulated in monocytes from IFNb non-responders compared with responders (Figure 4A).

For both ribosomal and immunoproteasomal pathways, gene expression signatures in monocytes from healthy controls were also closer to IFNb non-responders than to responders (Figures 3B and 4B).

## Discussion

Several lines of evidence support an important role of monocytes in MS pathogenesis: (i) circulating monocytes from MS patients are more active and they are present in brain active lesions [13]; (ii) monocytes from MS patients secrete more quantity of hydrogen peroxide and superoxide [14] and express more proinflammatory cytokines (TNFα, IL1β and IL6) during acute disease relapses upon stimulation [15]; (iii) MS patients have a higher percentage of monocytes secreting IL6 and IL12, and IL12-secreting monocytes are associated with higher disease activity when measured by magnetic resonance imaging [16]; (iv) non-classical type CD14+CD16+ (CCR5+) monocytes go through the blood brain barrier during acute phases of neuroinflammation [17].

A pharmacogenomic study from van Baarsen et al. reported negative significant correlations between baseline expression levels of type I IFN responsive genes and the biological responses measured by the induction ratios observed for these genes after 3 or 6 months of treatment with IFNb [18]. In a later study by our group, we identified a baseline over-expression of type I IFN responsive genes in PBMC from a subgroup of RRMS patients who showed a lack of response to IFNb [5], which was in part explained by the presence of an increased baseline expression of endogenous IFNb in non-responders [10]. Additional mechanistic experiments carried out by the group suggested a selective alteration of the type I IFN signaling pathway in monocytes from non-responders at baseline [5]: (i) increased STAT1 phosphorylation and IFNAR1 expression in monocytes but not in T cells, B cells, or myeloid or plasmacytoid dendritic cells; (ii) increased production of interferon-alpha (IFNa) upon TLR4 stimulation of PBMC. Based on these previous findings, our primary goal in the present study was to investigate whether type I IFN responsive genes were over-expressed in monocytes isolated from IFNb non-responders at baseline. To achieve this, CD14+ monocytes were first purified from PBMC of MS patients and healthy controls and then, gene expression profiling was determined with oligonucleotide microarrays. The clinical criteria used to classify patients into responders and non-responders to IFNb, was the same as in previous studies [5], [10]. The type I IFN signaling pathway appeared as one of the most differentially expressed pathways between responders and non-responders, and many type I IFN responsive genes were over-expressed in monocytes from IFNb non-responders when compared with responders. Furthermore, the transcription factor STAT1, a key mediator of the type I IFN signaling pathway, was also up-regulated in monocytes from non-responders. These results confirm previous findings by our group [5], [10], and altogether point out to a key role of blood monocytes as cell mediators of the therapeutic response to IFNb. In line with these results, in a recent study it was reported that the differential responses observed to *in vitro* stimulation with IFNb in PBMC cell subsets mainly lied in the monocyte subpopulation [12].

Evidence exists in the literature about the relevance of the type I IFN pathway in monocytes from patients with MS and other autoimmune disorders. Monocytes from MS patients was the only PBMC subset that increased TRAIL expression in response to IFNb treatment [12]. TRAIL is a proapoptotic transmembrane receptor induced by type I IFNs. It has been proposed that IFNb treatment predisposes monocytes to apoptosis through the induction of TRAIL [19]. Type I IFN signaling in monocytes may play an important role in the pathogenesis of MS since conditional genetic knockout of *ifnar1* in monocytes and neutrophils but not in other blood cell subtypes led to a more severe clinical course of EAE [11]. Additionally, a higher expression of type I IFN responsive genes was found in purified CD14+ monocytes from patients with Sjögren’s syndrome [9].

Another important goal in the present study was to identify additional biological pathways in monocytes that may also be playing important roles in the response to IFNb. Among the top canonical pathways that were differentially expressed between IFNb responders and non-responders, the mitochondrial dysfunction, eIF2 signaling and eIF4 and p70S6K signaling, and protein ubiquitinitation pathways were all considered potentially related with the response to IFNb.

A number of genes found to be differentially expressed pointed to mitochondrial dysfunction in monocytes from IFNb non-responders. Mitochondria generate ATP through the mitochondrial respiratory chain, which has important roles in processes such as cellular oxidation and apoptosis. In this context, the mitochondrial respiratory chain detects intra- and extra-cellular stresses and responds releasing citochrome c that leads to the activation of caspases and cell death. In our study, non-responders to IFNb treatment were characterized by an over-expression of genes belonging to the mitochondria respiratory chain, which could be due to the basal up-regulation of the type I IFN signaling pathway observed in these patients. Type I IFNs may modulate mitochondrial membrane stability and have apoptotic effects. For instance, in one study by Yanase et al. [20], type I IFNs were shown to induce mitochondrial depolarization, citochrome c release and caspase-3 activation in Daudi cells, a B-lymphoblastoid human cell line. In another study, Huang et al. [21] reported a clear interrelationship between the mitochondrial respiratory chain, IFN signaling and cell death. These authors observed synergistic effects of the retinoid acid and IFNb in increasing apoptotic processes mediated by mitochondria and a higher expression of mitochondrial respiratory chain genes [21].

IFNb has also been demonstrated to induce p53 signaling [22], [23]. Interestingly, a recent study by van Boxel-Dezaire et al. [24] pointed out to monocytes as the specific cell subset going into apoptosis after IFNb stimulation via activation of both STAT1 and STAT3. In our study, the transcription factors STAT1, p53 and DDIT3 (see Table S1) were all up-regulated in monocytes from non-responders, findings that, altogether, suggest increased apoptosis in monocytes from IFNb non-responders, most likely owing to mitochondrial depolarization.

A number of ribosomal genes were over-expressed in IFNb non-responders, and the eIF2 signaling and eIF4 and p70S6K signaling pathways, which are closely related with ribosomal protein synthesis, were significantly up-regulated in these patients. These findings may also be related with the baseline up-regulation of type I IFNs observed in non-responders. In this setting, type I IFNs can block protein synthesis by inducing the expression of the protein kinase PKR, which in turn phosphorylates eIF2 [25]. This translation initiation factor forms an inactivate complex with eIF2B to reduce protein synthesis within the cell. These processes are related with the antiviral functions of type I IFNs to inhibit viral transcription.

The protein ubiquitinitation pathway was up-regulated in monocytes from non-responders to treatment. Strikingly, when investigating genes related with this pathway, we observed that the vast majority of them belonged to the immunoproteasome machinery but not to the common proteasome. Immunoproteasome is highly involved in antigen presentation and monocytes are one of the antigen presenting cells of the immune system. The up-regulation of the ubiquitinitation pathway may also be related with the over-expression of type I IFNs in monocytes from non-responders. It has been reported that type I IFNs can induce the immunoproteasome switch [26], [27] and this effect seems to be long lasting producing a memory antiviral response [28]. In addition, some of the immunoproteasome-related genes that were up-regulated in non-responders such as TAP1, LMP2/PSMB9, and LMP7/PSMB8 are known to be induced by IFNb [29].

It is important to highlight that all these additional pathways found up-regulated in monocytes from IFNb non-responders are somehow related with type I IFN signaling, and prompt us to speculate that the over-expression of genes belonging to these pathways are a consequence of the baseline up-regulation of type I IFNs in this particular subgroup of patients. Finally, an intriguing finding that deserves some discussion is the behaviour of healthy controls in terms of gene expression, which is closer to IFNb non-responders than to responders for all the pathways that were considered relevant for treatment response. Similar findings in relation with type I IFN responsive genes were already reported in a previous study by our group [10], and may induce speculation about which is the aberrant group in the response to IFNb, “non-responders” or “responders”.

In summary, in the present study we confirm the baseline type I IFN signature in isolated monocytes from IFNb non-responders that was suggested from previous studies. Additional pathways relevant to monocyte biology and related to the IFNb effects were also up-regulated in monocytes from non-responders and, together with the type I IFN signaling pathway, may all be playing important roles in the response to IFNb.

## Materials and Methods

### Study Design and Clinical Assessment

In this study, RRMS patients treated with IFNb at the outpatient clinic of the *Centre d’Esclerosi Múltiple de Catalunya* (CEM-Cat) were included. All patients followed an internal protocol that consisted in collecting basal and longitudinal clinical data, as previously described [30]. All patients gave their informed consent and the study was approved by the local ethics committee. Clinical criteria of response to IFNb were applied after two years of treatment. Briefly, patients were classified as responders when there were no relapses and no increment in the EDSS (expanded disability status scale) score during the follow-up period. Patients were labeled as non-responders when there was one and more relapses and confirmed increase of at least one point in the EDSS score during the two years of follow-up [30]. Patients were labeled as intermediate responders when there was, during the follow-up period, presence of relapses with an increase of less than 1 point in the EDSS score, or absence of relapses with an increase in the EDSS score of one point or higher [10].

### Patients

Twenty RRMS patients and ten healthy controls were included in the study. Ten patients were classified as responders, 7 as non-responders, and 3 as intermediate responders to IFNb treatment. Insomuch as in a previous study we observed similar findings related with the type I IFN signature in stringent non-responders and intermediate responders [10], for the present study non-responders and intermediate responders were considered as a unique patient group labeled as “non-responders”. None of these patients had ever received treatment with IFNb or other immunosuppressive therapy before study entry. No patient had clinical exacerbations or received corticosteroid treatment during the month before initiation of IFNb. Table 2 summarizes the demographic and baseline characteristics of patients and healthy controls included in the study.

**Table 2:** Demographic and baseline clinical characteristics of MS patients and controls included in the study.

Characteristics	R	NR*	HC
n	10	10	10
Age (years)	32.5 (9.7)	34.7 (9.9)	28.2 (6.0)
Female**/**male (% women)	10/−(100)	7/3 (70)	8/2 (80)
Duration of disease (years)	2.6 (1.9)	5.7 (4.7)	–
EDSS^a^	2.0 (1.0–3.0)	2.4 (1.5–3.5)	–
Number of relapses^b^	2.3 (0.8)	2.1 (0.7)	–
Type of IFNb [n (%)]			
IFNb 1a IM	3 (30.0)	2 (20.0)	–
IFNb 1b SC	2 (20.0)	4 (40.0)	–
IFNb 1a SC	5 (50.0)	4 (40.0)	–

Data are expressed as mean (standard deviation) unless otherwise stated. ^a^Data are expressed as mean (interquartile range). ^b^Refers to the number of relapses in the two previous years. EDSS: Expanded Disability Status Scale. IM: intramuscular. SC: subcutaneous. R: responders to IFNb. NR: non-responders to IFNb. *NR includes 7 stringent non-responders and 3 intermediate responders to IFNb.

### Sample Collection and Monocyte Isolation

Peripheral blood was collected from healthy controls and RRMS patients before treatment with IFNb. PBMC were isolated by Ficoll-Isopaque density gradient centrifugation (Gibco BRL, Life Technologies LTD, UK) and stored in liquid nitrogen until used. For monocyte isolation, PBMC were thawed and stained with allophycocyanin (APC)-conjugated mouse anti-human CD14 (Pharmingen, San Diego, CA, USA). After cell washing, monocytes were separated based on their side-scatter pattern and CD14 positivity using a Legacy MoFlo Cell Sorter (Beckman-Coulter, Fullerton, CA, USA). Methodology of sorting was fit to achieve 99% of cell purity. The number of purified monocytes obtained per sample ranged from 80,000 to 800,000 cells.

### Total RNA Isolation and Pre-amplification

Total RNA was isolated from purified monocytes using the RNeasy Mini Kit (Qiagen, Germany). An additional step using DNase I (Quiagen, Germany) was included for DNA degradation. Total RNA was pre-amplified using the Ovation Pico WTA System (Nugen, CA, USA) according to the manufacturer’s recommendations. In brief, total RNA was used to synthesize single-stranded cDNA with random primers and subsequently converted into double-stranded cDNA, which was then used as template in an amplification reaction that resulted in numerous molecules of new single-stranded cDNA. The amplified single-stranded cDNA was finally purified using magnetic beads. Its quality, dimensions and concentration were checked in a Bioanalyzer 2100 (Agilent Technologies, CA, USA).

### Gene Expression Microarrays

Five µg of purified single-stranded cDNA was labeled and fragmented with the Ovation Biotin Module (Nugen, CA, USA) and their quality was checked in a Bioanalyzer 2100. The fragmented samples were then added to a hybridization cocktail containing Control oligonucleotide B2 (50 pM) and Eukaryotic Hybridization controls (BioB, BioC, BioD, cre) at 1.5, 5, 25 and 100 pM final concentration respectively from the GeneChip Eukaryotic Hybridization Control Kit (Affymetrix, CA, USA), herring sperm DNA (0.1 mg/ml) and acetylated BSA (0.5 mg/ml). Probe array was equilibrated to room temperature and prehybridized with 1× hybridization buffer (100 mM MES, 1 M [Na+], 20 mM EDTA, 0.01% Tween 20) at 45°C for 10 minutes with rotation. One hundred µl of the mixture were used for the hybridization to the Affymetrix Human Genome U219 (Affymetrix Genechip® array, CA, USA), that was performed at 60°C during 16 hours in a GeneTitan platform. This platform automates the hybridization, washing and scanning processes of arrays. GeneChips were washed and marked with streptavidin-phycoerythrin using the protocol EukGE-WS2-v5 provided by Affymetrix. Afterwards, they were scanned and processed in the Affymetrix software AGCC. Figure S1 illustrates the different steps undertaken in sample preparation and microarray processing.

### Data Analysis

All chips were one by one explored and checked for quality. All except one array passed through the quality control procedure. The signal distribution histograms (logarithmic) for the arrays were very similar with a peak of distribution falling into a 1.5–2.5 interval, while for the discarded array this peak was shifted to a 2.5–4.5 interval. The probeset-level log-scaled robust multiarray analysis (RMA) was performed with the Affymetrix Expression Console software. Linear models for microarray data R package was used to identify differentially expressed genes between responders and non-responders to IFNb treatment [31]. Differentially expressed genes (DEG) with p values<0.01 following two sample t tests were analyzed with the Ingenuity Pathway Analysis (IPA) software. The Euclidean distance between non-responders, responders and healthy controls was calculated according to the following formula:
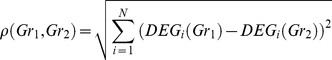



The d (Gr_1_,Gr_2_) is the calculated Euclidean distance. We summarized the data for a particular list of DEG displayed on each heatmap. DEG_i_ (Gr_N_) represents the particular mean value of gene expression level in the corresponding group.

Regarding data interpretation, we first compared DEG between responders and non-responders to IFNb. Gene expression profiling in responders and non-responders was subsequently compared to the healthy control group that was taken as a reference to determine the treated group that deviated from the healthy condition.

The Ingenuity Pathway Analysis was used to identify relevant differentially expressed pathways in monocytes from IFNb-treated patients. For pathway identification, only genes with p-values<0.01 were included in the analysis. For heatmap representation, differentially expressed genes with p-values<0.05 were considered. Insomuch as not all of the genes from the corresponding groups are annotated in the Ingenuity knowledge database, additional functionally related genes with p-values below <0.05 were also added to the heatmaps. P-values and fold changes for all the genes included in the pathways are provided in Table S2.

## Supporting Information

Figure S1 **Sample preparation and microarray processing workflow.** First steps involve PBMC extraction and monocyte isolation by cell sorting. Afterwards, RNA is extracted, retrotranscribed, and amplified to generate single strand cDNA (sscDNA). In subsequent steps, single-stranded cDNA is labeled, fragmented and hybridized to U219 expression arrays. As final steps, microarray data are analyzed and interpreted.
(TIF)

Table S1 **Differentially expressed genes, pathways and regulation genes.** In the first tab, the list of all differentially expressed genes between responders and non responders with raw data, fold change and p-values are represented (p<0.05). In the second tab, the list of all pathways returned by IPA is represented. In the third tab, all regulatory genes identified by IPA that were differentially expressed between responders and non responders are listed.
(XLS)

Table S2 **Genes included in heatmaps.** All genes included in heatmaps are listed in the table. Raw data for each group, p-values and fold changes (FC) are included.
(XLS)
