# Supplementary figures and images for: Baseline Gene Expression Signatures in Monocytes from Multiple Sclerosis Patients Treated with Interferon-beta
Source: PLoS One. 2013 Apr 18;8(4):e60994. doi: 10.1371/journal.pone.0060994 (PMC3630153; doi:10.1371/journal.pone.0060994)

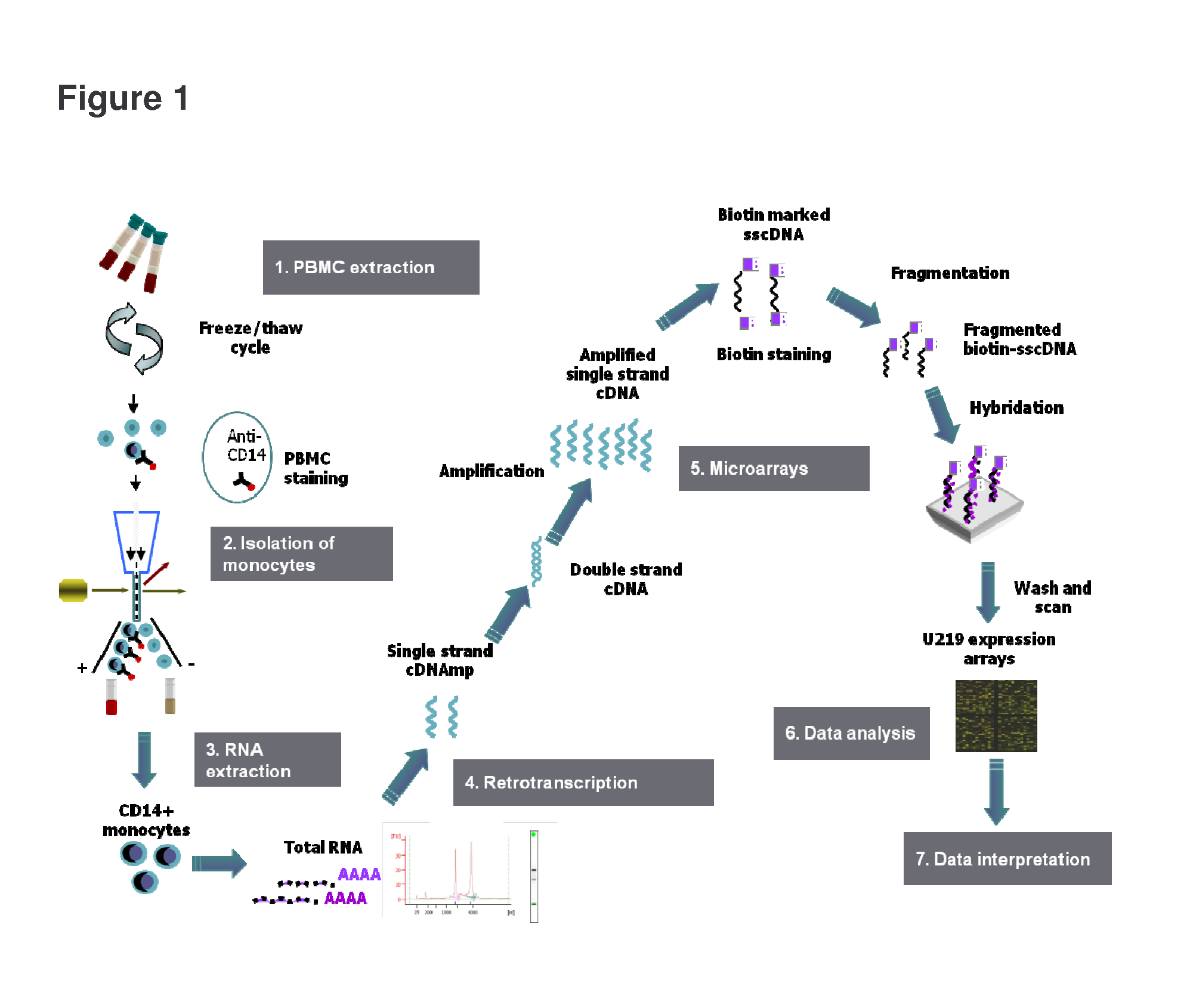

Supplement: Figure S1 — Sample preparation and microarray processing workflow. First steps involve PBMC extraction and monocyte isolation by cell sorting. Afterwards, RNA is extracted, retrotranscribed, and amplified to generate single strand cDNA (sscDNA). In subsequent steps, single-stranded cDNA is labeled, fragmented and hybridized to U219 expression arrays. As final steps, microarray data are analyzed and interpreted. (TIF) [file pone.0060994.s001.tif]
